# Body Composition and Cardiometabolic Risk Markers in Children of Women who Took Part in a Randomized Controlled Trial of a Preconceptional Nutritional Intervention in Mumbai, India

**DOI:** 10.1093/jn/nxab443

**Published:** 2022-01-07

**Authors:** Sirazul Ameen Sahariah, Meera Gandhi, Harsha Chopra, Sarah H Kehoe, Matthew J Johnson, Chiara di Gravio, Deepak Patkar, Harshad Sane, Patsy J Coakley, Aarti H Karkera, Dattatray S Bhat, Nick Brown, Barrie M Margetts, Alan A Jackson, Kalyanaraman Kumaran, Ramesh D Potdar, Caroline H D Fall

**Affiliations:** Centre for the Study of Social Change, Mumbai, India; Centre for the Study of Social Change, Mumbai, India; Centre for the Study of Social Change, Mumbai, India; Medical Research Council Lifecourse Epidemiology Centre, University of Southampton, Southampton, United Kingdom; Medical Research Council Lifecourse Epidemiology Centre, University of Southampton, Southampton, United Kingdom; Medical Research Council Lifecourse Epidemiology Centre, University of Southampton, Southampton, United Kingdom; Nanavati Hospital, Mumbai, India; Centre for the Study of Social Change, Mumbai, India; Medical Research Council Lifecourse Epidemiology Centre, University of Southampton, Southampton, United Kingdom; Nanavati Hospital, Mumbai, India; Diabetes Unit, KEM Hospital, Pune, India; International Center for Maternal and Child Health, Uppsala University, Uppsala, Sweden; Public Health Nutrition, University of Southampton, Southampton, United Kingdom; National Institute of Health Research, Biomedical Research Centre, Southampton, United Kingdom; Medical Research Council Lifecourse Epidemiology Centre, University of Southampton, Southampton, United Kingdom; Epidemiology Research Unit, CSI Holdsworth Memorial Hospital, Mysore, South India; Centre for the Study of Social Change, Mumbai, India; Medical Research Council Lifecourse Epidemiology Centre, University of Southampton, Southampton, United Kingdom

**Keywords:** maternal micronutrient supplementation, randomized controlled trial, India, children's body composition, children's glucose, children's insulin, children's lipids, DOHaD

## Abstract

**Background:**

Maternal nutrition influences fetal development and may permanently alter (“program”) offspring body composition and metabolism, thereby influencing later risk of diabetes and cardiovascular (cardiometabolic) disease. The prevalence of cardiometabolic disease is rising rapidly in India.

**Objectives:**

To test the hypothesis that supplementing low-income Indian women with micronutrient-rich foods preconceptionally and during pregnancy has a beneficial impact on the children's body composition and cardiometabolic risk marker profiles.

**Methods:**

Follow-up of 1255 children aged 5–10 y whose mothers took part in the Mumbai Maternal Nutrition Project [Project “SARAS”; International Standard Randomised Controlled Trial Number (ISRCTN)62811278]. Mothers were randomly assigned to receive a daily micronutrient-rich snack or a control snack of lower micronutrient content, both made from local foods, in addition to normal diet, from before pregnancy until delivery. Children's body composition was assessed using anthropometry and DXA. Their blood pressure, plasma glucose, insulin, and lipid concentrations were measured. Outcomes were compared between allocation groups with and without adjustment for confounding factors.

**Results:**

Overall, 15% of children were stunted, 34% were wasted, and 3% were overweight. In the intention-to-treat analysis, there were no differences in body composition or risk markers between children in the intervention and control groups. Among children whose mothers started supplementation ≥3 mo before conception (the “per protocol” sample) the intervention increased adiposity among girls, but not boys. BMI in girls was increased relative to controls by 2% (95% CI: 1, 4; *P* = 0.01); fat mass index by 10% (95% CI: 3, 18; *P* = 0.004); and percent fat by 7% (95% CI: 1, 13; *P* = 0.01) unadjusted, with similar results in adjusted models.

**Conclusions:**

Overall, supplementing women with micronutrient-rich foods from before pregnancy until delivery did not alter body composition or cardiometabolic risk markers in the children. Subgroup analyses showed that, if started ≥3 mo before conception, supplementation may increase adiposity among female children.

## Introduction

Ischemic heart disease (IHD) and type 2 diabetes (T2DM) are leading causes of disability and death worldwide (1). Although mortality from IHD is falling in the UK and other high-income countries, a trend attributed to both a falling incidence and improving medical treatment, it is increasing in low- and middle-income countries (LMICs) (1). The prevalence of T2DM is rising in all countries, along with obesity, but the most rapid increases are in LMICs, despite relatively low obesity rates (2, 3).

Around 30 y ago Barker, Hales, and others showed in a series of birth cohort studies that lower birth weight is associated with a higher risk of IHD and T2DM in adult life (4, 5). They proposed that fetal undernutrition is an important risk factor for cardiometabolic disease in later life, due to impaired development of metabolic tissues such as the pancreas, liver, kidneys, and skeletal muscle (6, 7); this became known as the “fetal programming” hypothesis. The same cohort studies showed that the highest risk of disease occurs in people who were small at birth but later became overweight (5, 8). This led to the concept that suboptimal fetal development results in reduced “metabolic capacity” throughout life, which leads to disease at a lower threshold of “metabolic load,” for example from later life obesity (9). This could explain high rates of cardiometabolic disease, out of proportion to current obesity levels, in LMICs where maternal undernutrition and low birth weight remain common problems (9).

Animal experiments, showing that undernourishing mothers leads to both fetal growth restriction and adult hypertension and diabetes in the offspring, support the fetal programming concept (10, 11). However, evidence for developmental programming in humans is still largely based on observational studies. Randomized controlled trials (RCTs) of nutritional interventions in undernourished women during pregnancy have shown that protein-energy and/or micronutrient supplements increase birth weight (12, 13). Follow-up of the children has shown reductions in blood pressure (14), fasting glucose (15), insulin resistance (16), LDL cholesterol (15), triglyceride concentrations (17), arterial stiffness (16), metabolic syndrome (17), and adiposity (18). However, these changes have been small, inconsistent across studies, and sometimes transient (19, 20) and some studies showed no beneficial effect on cardiometabolic outcomes (21, 22). Most of these trials started the nutritional intervention between 12 and 20 weeks of gestation, which would have missed events in early pregnancy that are potentially important for programming, such as placental development, the period of rapid fetal organogenesis, and periconceptional epigenetic changes (23).

The Mumbai Maternal Nutrition Project [Project “SARAS”, International Standard Randomised Controlled Trial Number [ISRCTN]62811278] was an RCT of a food-based micronutrient supplement, starting preconceptionally, for women from low-income families living in slum communities in Mumbai, India (24). The intervention was a daily snack made from micronutrient-rich local foods as a supplement to the women's normal diet. It reduced the incidence of gestational diabetes (25) and among women who started the supplement ≥3 mo prior to conception, increased birth weight, with larger effects among women who had a higher preconception BMI (24). We have now followed up the children to measure body composition and cardiometabolic risk markers at the age of 5–10 y. We hypothesized that the children of women in the intervention group would have lower cardiometabolic risk markers (blood pressure, serum lipids, plasma glucose, insulin resistance), and a healthier body composition (greater height and lean mass and lower body fat%) than children of mothers in the control group.

## Methods

### The trial

Project SARAS was a nonblinded individually randomized nutritional supplementation trial among women who were recruited before pregnancy in 2006–2011 (ISRCTN62811278) (24). The intervention was a daily snack made fresh each day in a trial kitchen from local micronutrient-rich vegetarian foods (green leafy vegetables, fruit, and milk) (26). Control snacks contained foods of lower micronutrient content (e.g. potato and onion). The aim was for women to take one snack every alternate day or more, for ≥3 mo before conception, and throughout pregnancy. On average, intervention snacks contained 10–23% of the WHO Reference Nutrient Intake (RNI) for β-carotene, riboflavin and vitamin B-12, folate, calcium, and iron, and 0.7 MJ of energy and 6 g of protein, compared with 0–7% RNI for the micronutrients, 0.4 MJ of energy, and 2 g of protein in control snacks (26, 27). Women were offered one snack daily; intake was supervised and the amount eaten was recorded (none, at least half, all). Women who became pregnant continued supplementation until delivery. All women were prescribed daily iron (100 mg) and folic acid (500 μg) supplements from the diagnosis of pregnancy, as per Indian government guidelines (28). Data were analyzed according to “intention-to-treat” (all women randomly assigned) and in the “per-protocol” subset of women who started supplementation >3 mo before conception. Of 6513 women recruited, 2291 became pregnant, leading to 1962 live singleton deliveries between 2007 and 2012.

### Children's follow-up

The “SARAS KIDS” follow-up study took place in 2013–2018, when the children were aged 5–10 y. Ethics approval was obtained from the Intersystem Biomedica Ethics Committee, Mumbai (ISBEC/NR-54/KM/JVJ/2013). Community health workers recontacted families by telephone or home visit, explained the study, and invited parents and children to attend a local clinic for investigations. Informed parental consent and the children's assent were obtained. All investigations were carried out on 1 d except for blood samples, which were done on a separate day after an overnight fast.

#### Anthropometry

Weight was measured once to the nearest 10 g using digital scales (ATCO Ltd). Height was measured once to the nearest millimeter using a wall-mounted stadiometer (Microtoise, CMS Instruments). Head and midupper-arm circumferences were each measured 3 times to the nearest millimeter using anthropometric tapes, and the mean value used in the analysis. Biceps, triceps, subscapular, and supra-iliac skinfolds were each measured to the nearest millimeter 3 times using Holtain skinfold callipers (CMS Instruments), and the mean value used in the analysis.

#### Blood pressure and pulse rate

Systolic and diastolic blood pressures and pulse rate were measured using an Omron 705IT digital monitor. Three measurements were made after the child had been seated for 5 min, removing and reapplying the cuff between measurements. The 3 values were averaged for analysis.

#### Plasma glucose, insulin, and lipids

Parents were asked to ensure that the child had nothing to eat or drink except water for ≥8 h before blood sampling. They were supplied with lidocaine anesthetic cream and shown how to apply this to the venepuncture site before leaving home. Fasting venous blood samples were taken for plasma glucose and insulin and serum lipid concentrations and additional samples were taken at 30 min after an oral glucose load of 1.75 g/kg anhydrous glucose dissolved in 300 mL of water for glucose and insulin, and after 120 min for glucose. Samples were placed on ice and centrifuged at 559 × *g* at room temerature for 25 minutes within 1 h. Plasma glucose concentrations were measured in a commercial laboratory (Dr Dharap's Laboratory, Dadar, Mumbai) using the glucose oxidase and peroxidase method, on the day of collection, using Accurex kits (Accurex Biomedical Ltd) and an EM200 autoanalyzer (Transasia Biomedicals Ltd). Plasma insulin and lipid samples were stored at –80°C and assayed at the end of the study in the laboratory of the Diabetes Unit, King Edward Memorial Hospital, Pune. Insulin was measured using ELISA kits (Mercodia AB, SE-754 50) and a Victor X4 multilabel plate reader (Perkin Elmer Life Sciences); their detection limit is 1.0 mU/L (ISO11843-Part-4). The kit is calibrated against the 1st International Reference Preparation 66/304. Inter- and intra-assay CVs were <7%. All the lipids were measured on an automated analyzer (Dialab) using Dialab ready-to-use kits. LDL cholesterol was measured by a 2-step enzymatic selective protection method. HDL cholesterol was measured using a homogeneous method without centrifugation steps; antibodies against human lipoproteins form antigen-antibody complexes with LDL, VLDL, and chylomicrons in a way that only HDL cholesterol is selectively determined by an enzymatic measurement. Triglycerides were measured using standard enzymatic kits. Inter- and intrabatch CVs for all 3 lipid measurements were <5%. Insulin sensitivity (HOMA-S) was estimated using the iHOMA2 online calculator (29). The insulogenic index {ln[Insulin(30-min/fasting)/Glucose (30-min/fasting)]} and the product of insulogenic index and insulin sensitivity, calculated as (insulogenic index + ln HOMA-S) were calculated as measures of pancreatic β-cell function.

#### Body composition

Whole-body and regional fat and lean mass were measured using DXA. Scans were carried out at the Department of Radiology, Nanavati Hospital, Vile Parle, Mumbai on a Lunar Prodigy fan beam DXA scanner, using pediatric software. The machine was calibrated daily according to the manufacturer's instructions. Hand grip strength was measured as a marker of muscle function using a Jamar dynamometer. Three measurements were made with each hand, and the maximum value used in the analysis.

#### Socioeconomic status

The family's socioeconomic status (SES) was assessed using the Standard of Living Index (SLI) questionnaire, developed for India's National Family Health Survey, which creates a score based on the size and quality of housing and amenities and ownership of land and household assets; a higher score reflects higher SES (30).

#### Definitions

Height and BMI were converted into *z*-scores based on the WHO 2007 standard (31, 32). Stunting was defined as a height-for-age <2 SDs below the WHO median. BMI-for-age was categorized as wasting if <2 SD below the WHO median; “normal” if between –2 and +1 SD of the WHO median; and overweight/obese if >1 SD above the WHO median.

### Statistical methods

Descriptive data are presented as mean ± SD for continuous normally distributed variables, median (IQR) for skewed variables, and *n* (%) for categorical variables. We tested the representativeness of the study sample by comparing maternal and newborn characteristics of: *1*) the children studied with the remainder of births in the original trial and *2*) within the study sample, between intervention and control groups. For comparisons of child outcomes between allocation groups, skewed variables were log-transformed, and all variables (except WHO *z*-scores and their derivatives) were adjusted for the child's age and sex. They were compared using 2-sided 2 sample *t*-tests for continuous variables and chi-square tests for categorical variables in: *1*) the full intention-to-treat sample and in *2*) the per protocol subgroup. We tested for interactions between allocation group and maternal prepregnant BMI and height as continuous variables, and the child's sex. Differences between groups are presented as mean difference and 95% CIs for normal continuous variables, as a multiplicative difference for log-transformed variables, and as ORs for binary outcomes. Statistical significance was set at *P* < 0.005 for comparisons of outcomes between intervention and control groups, using the Bonferroni correction for multiple testing and based on 10 “families” of outcomes (height, adiposity, lean/muscle, blood pressure, 3 lipid variables, glucose, and indices of insulin sensitivity and secretion). We used a significance level of *P* < 0.05 for interaction tests. Significant differences in outcomes between allocation groups were further examined using multiple linear regression, adjusting for potential confounders including maternal age, BMI, height, and parity, SES, and the child's birth weight and gestation. Kernel density plots were used to examine and compare the distribution of selected variables between allocation groups. Analyses were carried out using Stata SE v16.1 (33) and R v3.6.0 (34).

## Results

**FIGURE 1:**
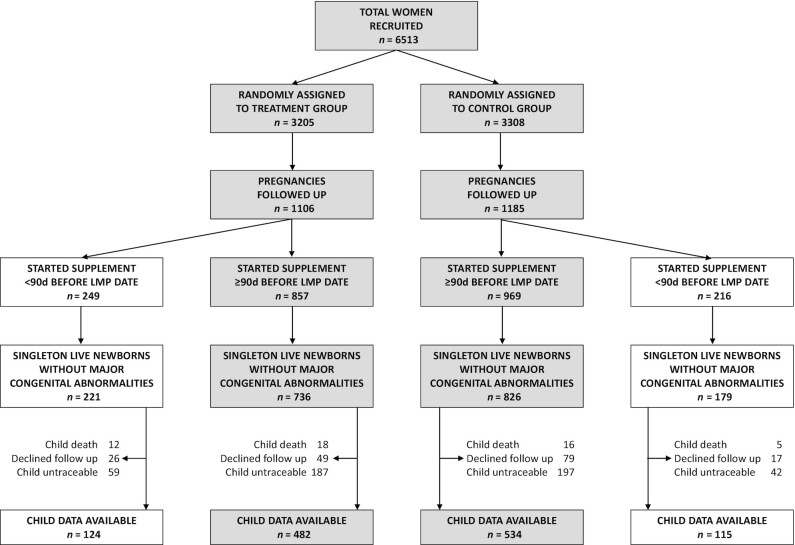
Participant flowchart. LMP, last menstrual period.

Of the 1962 live singleton births in the trial, 51 children died, 485 could not be retraced, and 171 declined to take part in the follow-up, leaving 1255 children (66% of survivors) who were studied (**Figure 1**). Their height- and BMI-for-age were low (mean WHO *z*-scores –1.0 and –1.5, respectively); 18% of boys and 13% of girls were stunted and 34% of boys and girls were wasted. Only 4% of boys and 2% of girls were overweight or obese (**Table 1**). Girls were more adipose than boys, whereas boys had a higher lean mass and grip strength. Boys had higher systolic blood pressure, fasting glucose concentration, and insulin sensitivity, whereas girls had a higher pulse rate, LDL cholesterol, and triglycerides (Table 1). Children studied were similar to those who were lost to follow-up in the proportions in each allocation group, maternal prepregnancy height, BMI, and gestational diabetes status, birth weight and sex ratio, but their mothers were older and of higher SES (**Table 2**). Among the children studied, maternal age, height, and Standard of Living Index (SLI) score were similar between the control and intervention groups, whereas maternal BMI was slightly lower in the intervention group (Table 2).

**FIGURE 2:**
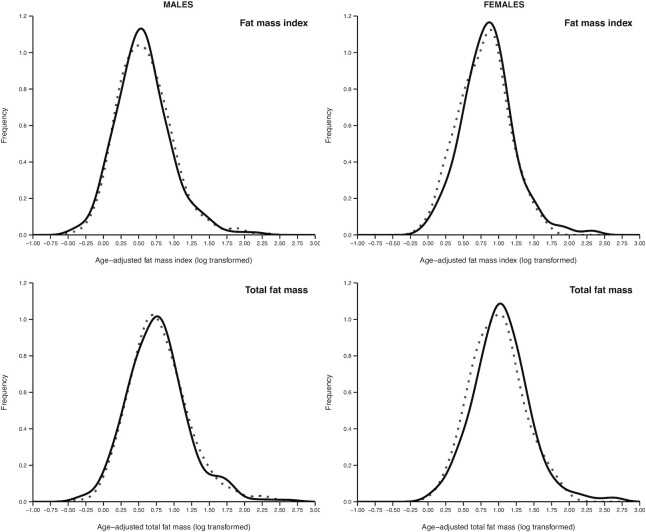
Kernel density plot of fat mass and fat mass index by intervention group in girls and boys.

**TABLE 1:** Characteristics of the study sample^1^

	Intention-to-treat sample (all children studied, maximum *n* = 1255)					Per protocol sample (children of mothers who started supplementation ≥3 mo before conception, maximum *n* = 1016)				
	Boys		Girls			Boys		Girls		
Outcome	*n*		*n*		*P* ^2^	*n*		*n*		*P^2^*
Anthropometry										
Height *z*-score	670	–1.0 ± 1.1	584	–1.0 ± 0.9	0.44	552	–1.0 ± 1.1	463	–1.0 ± 0.9	0.45
Stunted,^3^*n* (%)	670	118 (17.6)	584	76 (13.0)	0.03	552	98 (17.8)	463	61 (13.2)	0.05
BMI *z*-score	670	–1.5 ± 1.2	584	–1.6 ± 1.1	0.49	552	–1.5 ± 1.2	463	–1.6 ± 1.1	0.29
BMI categories,^3^*n* (%)										
Wasting	670	231 (34.5)	584	196 (33.6)	0.73	552	187 (33.9)	463	163 (35.2)	0.66
Normal BMI	670	414 (61.8)	584	377 (64.6)	0.31	552	344 (62.3)	463	292 (63.1)	0.81
Overweight/obese	670	25 (3.7)	584	11 (1.9)	0.05	552	21 (3.8)	463	8 (1.7)	0.05
Sum of skinfolds,^4^ mm	670	20.9 (18.2, 23.8)	584	23.3 (20.2, 26.8)	<0.001	552	20.8 (18.0, 23.8)	463	23.0 (20.0, 26.7)	<0.001
Grip strength, kg	669	7.0 ± 1.6	584	6.2 ± 1.4	<0.001	551	7.0 ± 1.6	463	6.2 ± 1.4	<0.001
Body composition (DXA)										
Fat mass,^4^ kg	660	2.1 (1.7, 2.8)	567	2.8 (2.1, 3.5)	<0.001	545	2.1 (1.7, 2.7)	450	2.8 (2.1, 3.4)	<0.001
Lean mass, kg	660	13.5 ± 1.6	567	12.3 ± 1.4	<0.001	545	13.5 ± 1.6	450	12.3 ± 1.4	<0.001
Percent fat,^4^ %	660	12.9 (10.6, 15.9)	567	17.2 (14.0, 20.5)	<0.001	545	13.0 (10.6, 15.9)	450	17.0 (13.9, 20.2)	<0.001
Cardiometabolic risk markers										
Systolic BP, mmHg	660	93.7 ± 8.6	583	91.9 ± 8.7	<0.001	543	93.8 ± 8.6	462	92.0 ± 8.6	<0.001
Diastolic BP, mmHg	660	56.4 ± 7.8	583	56.4 ± 7.0	0.95	543	56.4 ± 7.7	462	56.4 ± 7.1	0.94
Pulse rate, beats/min	667	95.7 ± 11.3	583	98.5 ± 11.5	<0.001	549	95.9 ± 11.1	462	98.7 ± 11.8	<0.001
LDL cholesterol, mmol/L	651	2.29 ± 0.61	559	2.42 ± 0.67	<0.001	535	2.29 ± 0.59	444	2.43 ± 0.64	<0.001
HDL cholesterol, mmol/L	651	1.08 ± 0.23	560	1.06 ± 0.22	0.17	535	1.07 ± 0.23	445	1.07 ± 0.22	0.62
Triglycerides,^4^ mmol/L	651	0.82 (0.68, 1.02)	560	0.88 (0.72, 1.11)	0.01	535	0.82 (0.68, 1.01)	445	0.88 (0.72, 1.11)	0.01
Fasting glucose, mmol/L	660	4.72 ± 0.56	572	4.62 ± 0.54	0.001	544	4.74 ± 0.54	454	4.62 ± 0.54	<0.001
120-min glucose, mmol/L	634	4.59 ± 0.87	549	4.73 ± 1.05	0.01	521	4.60 ± 0.84	434	4.73 ± 0.95	0.03
HOMA-S^4^	644	238 (151, 422)	552	207 (126, 323)	<0.001	528	244 (153, 414)	437	207 (129, 347)	0.004
Insulogenic index	639	1.7 ± 1.1	545	1.5 ± 1.2	0.003	525	1.7 ± 1.1	434	1.5 ± 1.2	0.004
Disposition index	632	7.1 ± 1.7	538	6.7 ± 1.8	<0.001	518	7.1 ± 1.6	427	6.7 ± 1.8	0.001

1 Values are mean ± SD unless otherwise specified. All body composition and cardiometabolic outcomes were adjusted for the child's age except for *z*-scores and BMI categories. HOMA-S: insulin sensitivity by Homeostasis Model Assessment.

2 P value denotes the significance of differences between boys and girls

3 Categorical variables are expressed as number *n*and (%).

4 Skewed variables are expressed as median and (IQR).

BP: Blood pressure.

**TABLE 2:** Representativeness of the study sample: maternal and newborn characteristics for the children included in the study sample compared with those lost to follow-up and, within the study sample, compared between maternal allocation groups^1^

	Included in this study maximum *n* = 1255		Lost to follow-up maximum *n* = 707		
Variable	*n*		*n*		*P* ^2^
Among all live singleton births in the original trial:					
Allocation group,^3^*n* (%)					
Control	649	(51.7)	356	(50.4)	0.56
Intervention	606	(48.3)	351	(49.6)	
Maternal age, y	1255	24.5 ± 3.9	707	23.5 ± 3.4	<0.001
Maternal height, cm	1255	151.3 ± 5.5	706	151.6 ± 5.4	0.19
Maternal prepregnancy BMI,^4^ kg/m^2^	1254	19.8 (17.8, 22.6)	706	19.8 (17.9, 22.1)	0.64
Maternal SLI score	1221	25.7 ± 5.7	683	23.7 ± 6.4	<0.001
Maternal GDM status,^3^*n* (%)					
No GDM	660	(89.8)	230	(91.3)	0.50
GDM	75	(10.2)	22	(8.7)	
Child's birthweight, g	960	2611 ± 381	407	2611 ± 419	0.92
Child's sex,^3^*n* (%)					
Male	671	(53.5)	233	(53.2)	0.53
Female	584	(46.5)	205	(46.8)	
Among children studied in this follow-up:	Control group maximum *n* = 649		Intervention group maximum *n* = 606		
Maternal age, y	649	24.7 ± 3.9	606	24.4 ± 3.8	0.17
Maternal height, cm	649	151.2 ± 5.4	606	151.3 ± 5.6	0.91
Maternal prepregnancy BMI,^4^ kg/m^2^	649	19.9 (17.9, 22.6)	606	19.6 (17.7, 22.5)	0.04
Maternal SLI score	629	25.7 ± 5.6	592	25.7 ± 5.8	0.90
Maternal GDM status,^3^*n* (%)					
No GDM	336	(87.7)	324	(92.1)	0.05
GDM	47	(12.3)	28	(8.0)	
Child's birth weight, g	499	2594 ± 393	461	2629 (368)	0.11
Child's sex,^3^*n* (%)					
Male	282	(56.3)	248	(53.6)	0.40
Female	219	(43.7)	215	(46.4)	

1 Values are mean ± SD unless otherwise specified.

2 P values denote the significance of differences between the groups shown

3 Categorical variables are expressed as number n and (%).

4 Skewed variables are expressed as median and (IQR).

GDM, maternal gestational diabetes mellitus; SLI: Standard of Living Index.

### Effect of the intervention

There were no significant differences in any of the outcomes between children whose mothers were in the control and intervention groups, in either the intention-to-treat or per protocol samples (**Table 3**). The results were similar when the sample was limited to women who were fully adherent with supplementation (**Supplemental Table 1**).

**TABLE 3:** Outcomes at 5–10 y according to allocation group^1^

	Intention-to-treat sample (all children studied, maximum *n* = 1255)						Per protocol sample (children of mothers who started supplementation ≥3 mo before conception, maximum *n* = 1016)					
	Control group		Intervention group				Control group		Intervention group			
Outcome	*n*		*n*		*P* ^1^	*P* ^2^	*n*		*n*		*P* ^1^	*P* ^2^
Anthropometry												
Height *z*-score	649	–1.0 ± 1.0	605	–1.0 ± 1.0	0.78	0.72	534	–1.0 ± 1.0	481	–1.0 ± 1.0	0.91	0.88
Stunted, *n* (%)	649	97 (15.0)	605	97 (16.0)	0.60	—	534	85 (15.9)	481	74 (15.4)	0.82	—
BMI *z*-score	649	–1.6 ± 1.1	605	–1.5 ± 1.2	0.45	0.16	534	–1.6 ± 1.1	481	–1.5 ± 1.2	0.23	0.04
BMI categories,^3^*n* (%)												
Wasting	649	224 (34.5)	605	203 (33.6)	0.72	—	534	190 (35.6)	481	160 (33.3)	0.44	—
Normal BMI	649	411 (63.3)	605	380 (62.8)	0.85	—	534	332 (62.2)	481	304 (63.2)	0.74	—
Overweight/obese	649	14 (2.2)	605	22 (3.6)	0.12	—	534	12 (2.3)	481	17 (3.5)	0.22	—
Sum of skinfolds,^4^ mm	649	21.9 (19.1, 24.9)	605	22.0 (19.1, 25.5)	0.36	0.18	534	21.7 (19.0, 24.8)	481	21.8 (19.0, 25.4)	0.36	0.02
Grip strength, kg	648	6.7 ± 1.6	605	6.6 ± 1.5	0.63	0.88	533	6.7 ± 1.6	481	6.6 ± 1.5	0.87	0.88
Body composition (DXA)												
Fat mass,^4^ kg	637	2.4 (1.9, 3.1)	590	2.5 (1.9, 3.1)	0.40	0.06	525	2.3 (1.9, 3.0)	470	2.5 (1.9, 3.1)	0.31	0.01
Lean mass, kg	637	13.0 ± 1.5	590	13.0 ± 1.5	0.97	0.72	525	13.0 ± 1.5	470	13.0 ± 1.5	0.62	0.53
Percent fat,^4^ %	637	14.8 (12.0, 18.0)	590	15.0 (12.3, 18.2)	0.40	0.08	525	14.7 (11.9, 17.9)	470	14.9 (12.3, 18.0)	0.39	0.02
Cardiometabolic risk markers												
Systolic BP, mmHg	641	92.9 ± 8.4	602	92.8 ± 8.9	0.75	0.34	526	92.9 ± 8.5	479	93.1 ± 8.8	0.75	0.14
Diastolic BP, mmHg	641	56.2 ± 7.3	602	56.6 ± 7.4	0.28	0.60	526	56.1 ± 7.3	479	56.8 ± 7.5	0.14	0.28
Pulse rate, beats/min	646	97.7 ± 11.5	604	96.3 ± 11.3	0.03	0.46	531	98.1 ± 11.2	480	96.2 ± 11.5	0.01	0.44
LDL cholesterol, mmol/L	627	2.38 ± 0.65	583	2.31 ± 0.61	0.05	0.18	515	2.39 ± 0.63	464	2.32 ± 0.59	0.07	0.29
HDL cholesterol, mmol/L	627	1.07 ± 0.23	584	1.07 ± 0.22	0.69	0.14	515	1.07 ± 0.23	465	1.07 ± 0.23	0.71	0.14
Triglycerides,^4^ mmol/L	627	0.85 (0.68, 1.05)	584	0.84 (0.70, 1.07)	0.98	0.89	515	0.85 (0.69, 1.05)	465	0.84 (0.70, 1.07)	0.69	0.49
Fasting glucose, mmol/L	640	4.68 ± 0.52	592	4.67 ± 0.58	0.54	0.85	526	4.69 ± 0.52	472	4.69 ± 0.55	0.91	0.74
120-min glucose, mmol/L	610	4.66 ± 0.88	573	4.65 ± 1.04	0.89	0.74	500	4.65 ± 0.87	455	4.67 ± 0.92	0.82	0.65
HOMA-S^4^	619	223 (142, 387)	577	223 (137, 376)	0.56	0.71	507	225 (148, 403)	458	228 (137, 367)	0.45	0.74
Insulogenic index	614	1.63 ± 1.11	570	1.50 ± 1.16	0.06	0.66	507	1.66 ± 1.13	452	1.53 ±1.17	0.08	0.99
Disposition index	606	7.0 ± 1.7	564	6.9 ± 1.7	0.17	0.82	499	7.0 ± 1.7	446	6.9 ± 1.7	0.19	0.96

1 ^,2^Values are mean ± SD unless otherwise specified. All body composition and cardiometabolic outcomes were adjusted for the child's age and sex except for Z-scores. *P*1: significance of difference between control and intervention groups; *P*2: significance of interaction between allocation group and sex. BP: blood pressure; HOMA-S: insulin sensitivity by Homeostasis Model Assessment.

3 Categorical variables are expressed as number (*n*) and (%).

4 Skewed variables are expressed as median and (IQR).

In the per protocol sample, there were significant sex interactions (allocation group × child's sex) for the adiposity outcomes: BMI, skinfolds, and fat mass and percent fat measured by DXA (Table 3). Among girls only, these adiposity measures were higher in the intervention group (**Table 4**); BMI was increased by 2% (95% CI: 1, 4; *P* = 0.01); fat mass index by 10% (95% CI: 3, 18; *P* = 0.004); and percent fat by 7% (95% CI: 1, 13; *P* = 0.01). The prevalence of wasting was decreased, and that of normal BMI and overweight/obesity increased, though none of these effects were statistically significant (Table 4). Kernel density plots suggested an approximately symmetrical right shift in fat mass and fat mass index (**Figure 2**). Regression analysis, adjusting for confounding factors, showed that the increased adiposity among girls in the intervention group remained significant after adjusting for maternal characteristics, and may be partly influenced by the higher birth weight in the intervention group (shown for fat mass index in **Table 5** and for other adiposity measures in **Supplemental Table 2**). There were no interactions between allocation group and maternal BMI or height.

**TABLE 4:** Adiposity measurements in the children according to the mother's allocation group, stratified by sex (per protocol sample)^1^

	Boys				Girls			
Outcome	Control *n* = 299	Intervention *n* = 253	Difference (intervention-control)^2^ (95% CI)	*P* ^3^	Control *n* = 235	Intervention *n* = 228	Difference (intervention-control)^2^ (95% CI)	*P* ^3^
Anthropometry								
BMI,^4^ kg/m^2^	13.5 (12.8, 14.1)	13.4 (12.8, 14.0)	1.00 (0.98, 1.01)	0.62	13.0 (12.3, 13.9)	13.2 (12.6, 14.1)	1.02 (1.01, 1.04)	0.01
BMI *z*-score (WHO)	–1.5 ± 1.2	–1.5 ± 1.2	–0.0 (–0.2, 0.1)	0.62	–1.7 ± 1.1	–1.4 ± 1.2	0.3 (0.0, 0.5)	0.02
BMI categories,^5^*n* (%)								
Wasting	100 (33.4)	87 (34.4)	1.04 (0.73, 1.49)	0.82	90 (38.3)	73 (32.0)	0.76 (0.52, 1.11)	0.16
Normal BMI	189 (63.2)	155 (61.3)	0.92 (0.65, 1.30)	0.64	143 (60.9)	149 (65.4)	1.21 (0.83, 1.77)	0.32
Overweight/obese	10 (3.3)	11 (4.4)	1.31 (0.55, 3.15)	0.54	2 (0.9)	6 (2.6)	3.15 (0.63, 15.77)	0.14
Biceps skinfold,^4^ mm	4.4 (3.9, 5.3)	4.3 (3.9, 5.1)	0.98 (0.94, 1.02)	0.28	4.8 (4.1, 5.5)	5.0 (4.3, 6.0)	1.05 (1.01, 1.10)	0.02
Triceps skinfold,^4^ mm	6.9 (5.8, 7.9)	6.7 (5.8, 7.9)	0.99 (0.94, 1.03)	0.61	7.3 (6.1, 8.4)	7.6 (6.4, 9.1)	1.06 (1.01, 1.11)	0.01
Subscapular skinfold,^4^ mm	5.4 (4.8, 6.3)	5.3 (4.7, 6.3)	0.98 (0.94, 1.02)	0.25	5.9 (5.0, 6.7)	6.2 (5.4, 7.4)	1.07 (1.03, 1.12)	0.002
Suprailiac skinfold,^4^ mm	4.0 (3.3, 4.8)	3.9 (3.3, 4.6)	0.98 (0.94, 1.03)	0.53	4.6 (3.8, 5.5)	4.6 (4.0, 5.7)	1.02 (0.97, 1.07)	0.55
Sum of skinfolds,^4^ mm	20.8 (18.3, 24.2)	20.6 (17.8, 23.6)	0.98 (0.94, 1.02)	0.37	22.5 (19.7, 25.7)	23.3 (20.3, 28.0)	1.05 (1.01, 1.10)	0.01
Body composition (DXA)								
Fat mass,^4^ kg	2.13 (1.71, 2.83)	2.15 (1.63, 2.66)	0.97 (0.90, 1.04)	0.37	2.55 (2.01, 3.21)	2.86 (2.23, 3.62)	1.11 (1.03, 1.19)	0.01
Fat mass index,^4^ kg/m^2^	1.8 (1.4, 2.3)	1.7 (1.4, 2.2)	0.97 (0.91, 1.03)	0.32	2.2 (1.7, 2.7)	2.4 (1.8, 2.9)	1.10 (1.03, 1.18)	0.004
Lean mass, kg	13.5 ± 1.7	13.5 ± 1.7	–0.0 (–0.3, 0.3)	0.95	12.3 ± 1.4	12.4 ± 1.4	0.1 (–0.1, 0.4)	0.36
Lean mass index, kg/m^2^	11.0 ± 0.7	11.0 ± 0.7	0.0 (–0.1, 0.1)	0.99	10.1 ± 0.7	10.2 ± 0.7	0.1 (–0.1, 0.2)	0.26
Percent fat,^4^ %	13.0 (10.8, 16.3)	12.9 (10.4, 15.6)	0.98 (0.92, 1.03)	0.36	16.8 (13.6, 19.8)	17.2 (14.7, 20.6)	1.07 (1.01, 1.13)	0.01
Android fat,^4^ kg	0.15 (0.11, 0.20)	0.14 (0.11, 0.18)	0.98 (0.90, 1.06)	0.61	0.18 (0.13, 0.23)	0.19 (0.15, 0.25)	1.10 (1.01, 1.20)	0.03
Gynoid fat,^4^ kg	0.57 (0.48, 0.73)	0.56 (0.44, 0.67)	0.97 (0.91, 1.03)	0.26	0.69 (0.57, 0.81)	0.72 (0.60, 0.88)	1.06 (1.00, 1.12)	0.04

1 Values are mean ± SD unless otherwise specified. All outcomes are adjusted for the child's age.

2 Differences between allocation groups: for continuous normally distributed variables, these are expressed as raw values in the intervention group minus those in the control group, with 95% CIs. For skewed variables^3^, which were log-transformed for the analysis, the differences are exponentiated, and indicate the multiplicative difference between control and intervention groups; for example: a value of 1.07 means that the outcome was 7% higher in the intervention group than in the control group, whereas a value of 0.97 means that the outcome was 3% lower in the intervention group. For categorical variables^2^, the differences between groups are expressed as ORs, with the control group as the reference category.

3 P values denote the significance of differences between control and intervention groups.

4 Skewed variables are expressed as median and (IQR).

5 Categorical variables are expressed as number (*n*) and (%).

**TABLE 5:** Multiple linear regression analysis of allocation group as a predictor of log fat mass index in girls only, adjusted for maternal and newborn characteristics (per protocol sample)

	Model 1, *N* = 438			Model 2, *N* = 317		
Exposure	Linear regression coefficient	95% CIs	*P* value	Linear regression coefficient	95% CIs	*P* value
Maternal						
Allocation group (control = 0, intervention = 1)	0.104^1^	0.037, 0.170	0.003	0.085^1^	0.004, 0.167	0.04
Age, y	–0.009	–0.018, –0.000	0.05	–0.016	–0.028, –0.005	0.005
BMI, kg/m^2^	0.025	0.016, 0.034	<0.001	0.024	0.013, 0.036	<0.001
Height, cm	–0.004	–0.010, 0.002	0.20	–0.005	–0.012, 0.002	0.18
Parity – primiparous	–0.059	–0.139, 0.020	0.15	–0.054	–0.158, 0.050	0.31
Parity – multiparous	–0.112	–0.218, –0.005	0.04	–0.115	–0.243, 0.014	0.08
Socioeconomic status score	–0.000	–0.007, 0.006	0.91	–0.003	–0.010, 0.005	0.49
Child						
Age, y	0.053	0.006, 0.0101	0.03	0.032	–0.029, 0.093	0.31
Birth weight, kg	—	—	—	0.128	0.111, 0.245	0.03
Gestational age, weeks	—	—	—	–0.012	–0.035, 0.010	0.29

1 For allocation group, the regression coefficient represents the difference in log fat mass between the intervention and control groups. To translate this into a more meaningful value the coefficient is antilogged (exponentiated: values become 1.11 for Model 1 and 1.09 for Model 2) and this value indicates the multiplicative difference between control and intervention groups; for example: an exponentiated value of 1.11 means that fat mass index was 11% higher in the intervention group than in the control group.

## Discussion

### Summary of findings

This study examined the impact of a preconceptional maternal nutritional intervention in an RCT on cardiometabolic risk markers and body composition in the children. The intervention, a micronutrient-rich food supplement from before conception and throughout pregnancy, had no effect overall on the children's cardiometabolic risk markers or body composition. In the subgroup of children whose mothers started supplementation ≥3 mo before conception, girls had a higher BMI and were more adipose in the intervention group compared with controls.

### Cardiometabolic risk markers

Possible reasons for a lack of effect on risk markers are that: *1*) the intervention did not sufficiently improve maternal nutritional status; *2*) a nutritional intervention alone is not sufficient to improve fetal development among women living with multiple environmental stresses likely to influence outcomes (poverty, overcrowding, pollution, inadequate sanitation); *3*) a lack of obesity among these children meant that risk markers remained low in both groups; *4*) the children were too young to see an effect; or *5*) maternal diet and nutrition are not important influences on children's cardiometabolic risk markers. We chose a food-based supplement based on findings from the Pune Maternal Nutrition Study, and for greater acceptability and potential greater scalability in future, but the “dose” of micronutrients that it supplied was low (maximum 23% RNI) compared with other nutritional interventions used in trials, such as the United Nations International Multiple Micronutrient Preparation (UNIMMAP) tablet (100% RNI). In a separate study among nonpregnant women in Mumbai we showed that the SARAS supplement increased circulating β-carotene (35) and n–3 fatty acid concentrations (36), but did not significantly alter ferritin, retinol, ascorbate, folate, or vitamin B-12 status (35). Additionally, despite our efforts to make the snacks tasty and varied, it was challenging to sustain full adherence to supplementation over the long period of time required in a preconceptional trial. For these reasons, the effect of preconceptional nutritional supplementation requires testing in further trials, and it will be interesting to see the results of several completed or ongoing preconceptional trials which set out to deliver higher doses of multiple micronutrients in tablet or other ready-made form and achieved higher compliance rates (37–41). Ultimately, sustainable ways of improving diet quality, using food, will be necessary. Improved maternal diet on its own may not be sufficient to achieve optimal fetal development; in preventing childhood stunting, another widespread problem in LMICs caused by complex multiple exposures, combinations of interventions, targeting both health and nutrition outcomes, have proved most successful (42). That improved maternal nutrition alone may not be sufficient for optimal fetal development in the face of multiple environmental challenges is recognized in the ongoing HeLTI (Healthy Life Trajectory Initiative) and WINGS (Women and Infants Integrated Growth Study) randomized trials, which aim to improve maternal mental health as well as nutrition, and reduce infection and environmental pollution (43, 44).

### Body composition

Daughters of women in the intervention group who started supplementation well before conception (>3 mo) were more adipose than daughters of women in the control group. The trial was designed to test this group separately (24), the rationale being that we would expect around 3 mo of supplementation to be required to achieve its full impact on maternal nutritional status. The effect of the intervention on adiposity in girls was physiologically significant, at ∼10% increase in fat mass index. Overall, the prevalence of wasting was 34% among the study children, whereas that of overweight/obesity was only 3%, and increased adiposity may therefore indicate more optimal nutrition. Greater adiposity provides opportunity for better future childhood and pubertal statural growth and (in girls) later reproductive outcomes. However, a gain in body fat without concomitant gains in height and lean mass could also have adverse cardiometabolic effects in adult life. Greater adiposity could reflect advanced maturation; it was not possible to determine this, although the differences in adiposity between allocation groups were not greater at older ages. Some of these possibilities will become clear with further follow-up. Most previous trials (all starting in midpregnancy) using multiple micronutrients (14, 45, 46), protein energy (16, 22), or n–3 fatty acids (47–49) reported no increase in adiposity in the children. However, in 3 multiple micronutrient supplementation trials in Burkina Faso, Nepal, and Bangladesh, children of mothers who received multiple micronutrients had higher BMI or weight-for-height *z*-scores at age 30 mo, 8.5 y, and 9 y, respectively (50, 19, 51). In the Nepal trial, as in Mumbai, this effect was present only in girls (19). In the Burkina Faso trial the positive effect on weight-for-height was accompanied by an increase in height (50).

### Programming of adiposity

There is observational evidence in humans and interventional evidence in experimental animals that maternal *undernutrition* during pregnancy increases later adiposity in the children/offspring. Exposure of previously well-nourished women to the Dutch Famine in early gestation was associated with greater adult adiposity in their children, of both sexes (52, 53). In rats, both dietary restriction (either global restriction or a low-protein diet) and *overfeeding* of mothers during pregnancy increases adiposity in the adult offspring (11, 54–56). None of these dietary experiences or experimental manipulations remotely corresponds to our intervention in Mumbai (supplementation of mothers, many of whom were chronically undernourished, with physiological doses of micronutrient-rich foods) but they show that adipose tissue is “programmable” by maternal diet in pregnancy, including under- and overfeeding. Animal studies have shown that various mechanisms play a role in such experimental programming, including altered appetite (e.g. hyperphagia), food choices (e.g. junk food preference), reduced physical activity or resting energy expenditure, altered concentrations of or sensitivity to hormones (e.g. cortisol and leptin) or inflammatory markers, impaired mitochondrial function, altered mesenchymal stem cell commitment (to adipocyte as opposed to muscle/bone/cartilage lineages), and epigenetic changes (11, 54–56). Perhaps the closest animal experiment to our study was the “thrifty jerry” rat model, in which rats were globally undernourished for many generations, followed by recuperation onto normal feeding (57). During the undernourished phase, newborn pups were smaller than controls but became excessively adipose as adults. After a return to normal feeding (ad libitum standard chow) birth weight was restored to control levels, but adult adiposity remained, and exceeded that in the multigenerationally undernourished offspring. This was associated with epigenetic changes in the insulin-2 promoter region, which persisted after recuperation (57). Unlike our study, the increased adiposity among recuperated offspring was associated with elevated glucose, insulin, and lipid concentrations.

### Sex differences

There is extensive literature from experimental animals reporting sex differences in phenotypic outcomes in offspring following maternal nutritional deprivation or overfeeding (58, 59). For example in rats, maternal protein deprivation during pregnancy consistently leads to raised adult blood pressure in male but not female offspring. There are isolated examples of sex differences in the human developmental programming literature, but no consistent pattern has emerged linking particular exposures or outcomes to one or other sex (58, 59). Apart from the Nepal trial described above (19), none of the child follow-ups from maternal supplementation trials in pregnancy have reported sex differences in cardiometabolic or body composition outcomes, but only a minority formally tested for sex differences. Mechanisms underlying sex differences in developmental programming in animals are still poorly understood (58–60). The fact that we observed a sex difference in the effect on adiposity only in the per protocol sample of children suggests that the critical period of exposure was periconceptional or in very early pregnancy, possibly related to sex differences in periconceptional gene expression or epigenetic characteristics in the embryo or placenta (59). In rodents, both maternal nutrient restriction and overfeeding lead to sex-specific changes in DNA methylation in the placenta (60).

### Strengths and limitations

We studied a large sample of children, and cardiometabolic risk markers and body composition were measured using standard methods. A limitation was that we studied only 64% of the children born in the original trial. The greatest loss to follow-up was from families moving out of the study area, either through migration or relocation after local authority slum clearance. These losses were minimized by community health workers continually updating mobile phone numbers and attempting to retain contact with parents. We reimbursed families’ expenses to come to the clinic from the main relocation areas ∼20–30 km away. The children studied were similar to those lost to follow-up in key characteristics, but their mothers were older and of higher SES (Table 2). This could be because, in our experience, better-off families were more likely to own rather than rent their dwelling and therefore less likely to get moved out, and more likely to have a permanent mobile phone number. However, SES did not differ between allocation groups and our results were unchanged after adjusting for maternal age and SES and other potential confounding factors.

### Conclusions and implications

The intervention, a preconception and pregnancy daily snack made from micronutrient-rich local foods, which increased birth weight and reduced the incidence of gestational diabetes did not alter the children's cardiometabolic risk markers. Girls of mothers who started the intervention >3 mo before conception had a higher BMI, were less likely to be wasted, and were more adipose. We do not know the significance of this for future health outcomes and will continue to follow-up these children.

## Supplementary Material

nxab443_Supplemental_File

## Data Availability

Data described in the manuscript, code book, and analytic code will be made available upon request pending application and approval.

## Notes

The original trial was supported by grants from United States Agency for International Development (USAID), the Wellcome Trust, the Parthenon Trust, and ICICI Bank Ltd., Mumbai. The current study was funded by Medical Research Council Research Grant number: MR/M005186/1. None of the funders played a role in study design or implementation, or analysis and interpretation of data.

Author disclosures: The authors report no conflicts of interest.

Supplemental Tables 1 and 2 are available from the “Supplementary data” link in the online posting of the article and from the same link in the online table of contents at https://academic.oup.com/jn/.

Abbreviations used: HOMA, homeostasis model assessment; IHD, ischemic heart disease; ISRCT, International Standard Randomised Controlled Trial Number; LMIC, low- or middle-income country; T2DM, type 2 diabetes mellitus; RCT, randomized controlled trial; RNI, reference nutrient intake; SARAS, Mumbai Maternal Nutrition Project; SES, socioeconomic status; SLI, Standard of Living Index.
