# Supplementary material for: Body Composition and Cardiometabolic Risk Markers in Children of Women who Took Part in a Randomized Controlled Trial of a Preconceptional Nutritional Intervention in Mumbai, India
Source: J Nutr. 2022 Jan 7;152(4):1070–81. doi: 10.1093/jn/nxab443 (PMC8971001; doi:10.1093/jn/nxab443)
Supplement: nxab443_Supplemental_File [file nxab443_supplemental_file.docx]

**Sahariah SA et al. Body composition and cardiometabolic risk markers in children of women who took part in a randomized controlled trial of a pre-conceptional nutritional intervention in Mumbai, India**

**ONLINE SUPPLEMENTARY MATERIAL**

**For online publication only**

**Supplementary Table 1: Outcomes at 5-10 years according to allocation group among children whose mothers were fully compliant with supplementation ^1^**

|  | **INTENTION TO TREAT SAMPLE**  **(all children studied, Maximum N-1,255)** | | | | | | **PER PROTOCOL SAMPLE**  **(children of mothers who started supplementation >3m before conception, Maximum N=1,016)** | | | | | |
| --- | --- | --- | --- | --- | --- | --- | --- | --- | --- | --- | --- | --- |
| **Outcome** | **CONTROL GROUP** | | **INTERVENTION GROUP** | | **p^1^** | **p^2^** | **CONTROL GROUP** | | **INTERVENTION GROUP** | | **p^1^** | **p^2^** |
|  | **n** |  | **n** |  |  |  | **n** |  | **n** |  |  |  |
| **Anthropometry** | | | | | | | | | | | | |
| Height Z-score | 331 | **-1.0** (1.0) | 224 | **-1.1** (1.0) | **0.28** | **0.83** | 271 | **-1.1** (1.0) | 177 | **-1.1** (1.0) | **0.85** | **0.89** |
| Stunted ^2^ (N(%)) | 331 | **48** (14.5) | 224 | **38** (17.0) | **0.43** | **-** | 271 | **45** (16.6) | 177 | **28** (15.8) | **0.83** | **-** |
| BMI Z-score | 331 | **-1.6** (1.1) | 224 | **-1.5** (1.2) | **0.28** | **0.16** | 271 | **-1.6** (1.1) | 177 | **-1.4** (1.2) | **0.13** | **0.19** |
| BMI categories ^2^ (N (%)) |  |  |  |  |  |  |  |  |  |  |  |  |
| Wasting | 331 | **124** (37.5) | 224 | **78** (34.8) | **0.53** | **-** | 271 | **103** (38.0) | 177 | **59** (33.3) | **0.31** | **-** |
| Normal BMI | 331 | **201** (60.7) | 224 | **138** (61.6) | **0.83** | **-** | 271 | **163** (60.2) | 177 | **111** (62.7) | **0.59** | **-** |
| Overweight/obese | 331 | **6** (1.8) | 224 | **8** (3.6) | **0.20** | **-** | 271 | **5** (1.9) | 177 | **7** (4.0) | **0.18** | **-** |
| Sum of skinfolds ^3^ (mm) | 331 | **21.9** (19.3, 25.3) | 224 | **22.5** (19.6, 25.6) | **0.22** | **0.24** | 271 | **21.8** (19.0, 25.4) | 177 | **22.3** (19.6, 25.7) | **0.22** | **0.21** |
| Grip strength (kg) | 331 | **6.7** (1.7) | 223 | **6.4** (1.3) | **0.63** | **0.35** | 271 | **6.6** (1.6) | 176 | **6.4** (1.3) | **0.10** | **0.37** |
| **Body composition (DXA)** | | | | | | | | | | | | |
| Fat mass ^3^ (kg) | 323 | **2.4** (1.9, 3.1) | 218 | **2.5** (1.9, 3.1) | **0.40** | **0.27** | 265 | **2.3** (1.8, 3.1) | 172 | **2.5** (1.9, 3.1) | **0.28** | **0.25** |
| Lean mass (kg) | 323 | **12.9** (1.6) | 218 | **12.8** (1.5) | **0.48** | **0.22** | 265 | **12.9** (1.6) | 172 | **12.9** (1.5) | **0.73** | **0.21** |
| Fat % ^3^ | 323 | **14.8** (12.0, 17.9) | 218 | **15.2** (12.6, 18.5) | **0.27** | **0.52** | 265 | **14.8** (11.9, 17.8) | 172 | **15.0** (12.5, 18.5) | **0.30** | **0.44** |
| **Cardiometabolic risk markers** | | | | | | | | | | | | |
| Systolic BP (mmHg) | 325 | **92.6** (8.3) | 223 | **93.5** (8.7) | **0.21** | **0.40** | 265 | **92.8** (8.5) | 176 | **93.8** (8.9) | **0.23** | **0.33** |
| Diastolic BP (mmHg) | 325 | **55.8** (7.3) | 223 | **57.0** (7.2) | **0.05** | **0.59** | 265 | **55.8** (7.3) | 176 | **57.2** (7.3) | **0.05** | **0.30** |
| Pulse rate (beats/min) | 330 | **97.2** (11.7) | 223 | **97.6** (10.4) | **0.65** | **0.10** | 270 | **97.5** (11.6) | 176 | **97.6** (10.5) | **0.95** | **0.07** |
| LDL cholesterol (mmol/l) | 316 | **2.43** (0.63) | 214 | **2.29** (0.57) | **0.01** | **0.24** | 257 | **2.44** (0.60) | 169 | **2.30** (0.57) | **0.02** | **0.12** |
| HDL cholesterol (mmol/l) | 316 | **1.07** (0.21) | 214 | **1.08** (0.22) | **0.44** | **0.19** | 257 | **1.07** (0.21) | 169 | **1.08** (0.22) | **0.54** | **0.30** |
| Triglycerides ^3^ (mmol/l) | 316 | **0.84** (0.67, 1.03) | 214 | **0.86** (0.73, 1.10) | **0.19** | **0.95** | 257 | **0.83** (0.68, 1.02) | 169 | **0.86** (0.74, 1.11) | **0.17** | **0.86** |
| Fasting glucose (mmol/l) | 325 | **4.67** (0.53) | 217 | **4.68** (0.56) | **0.80** | **0.50** | 265 | **4.66** (0.53) | 172 | **4.70** (0.52) | **0.41** | **0.86** |
| 120-min glucose (mmol/l) | 312 | **4.63** (0.92) | 210 | **4.61** (0.95) | **0.88** | **0.86** | 254 | **4.62** (0.91) | 165 | **4.63** (0.93) | **0.89** | **0.72** |
| HOMA-S ^3^ | 311 | **220** (139, 446) | 214 | **224** (137, 387) | **0.75** | **0.15** | 252 | **222** (142, 446) | 169 | **231** (137, 376) | **0.69** | **0.53** |
| Insulogenic index | 309 | **1.6** (1.1) | 208 | **1.4** (1.1) | **0.05** | **0.97** | 252 | **1.6** (1.1) | 164 | **1.5** (1.1) | **0.25** | **0.96** |
| Disposition index | 304 | **7.0** (1.7) | 208 | **6.8** (1.7) | **0.32** | **0.44** | 247 | **7.0** (1.7) | 164 | **6.9** (1.7) | **0.66** | **0.66** |

^1^ Values are mean (SD) unless otherwise specified. All body composition and cardiometabolic outcomes were adjusted for the child’s age and sex except for Z-scores. P1: significance of difference between control and intervention groups; P2: significance of interaction between allocation group and sex.

^2^ Categorical variables are expressed as number (N) and percent.

^3^ Skewed variables are expressed as median and inter-quartile range.

DXA: Dual X-ray absorptiometry; HOMA-S: insulin sensitivity by Homeostasis Model Assessment.

**Supplementary Table 2: Differences in adiposity between control and intervention groups in girls, adjusted for potential confounding factors (per protocol sample) ^1^**

| **Outcomes** | **Linear**  **Regression coefficient for allocation group ^2^** | **Difference [intervention-control] (95% CI) ^3^** | **P** |
| --- | --- | --- | --- |
| **MODEL 1 ^4^** |  |  |  |
| **Anthropometry** |  |  |  |
| Body mass index (kg/m^2^) | 0.032 | 1.03 (1.01, 1.05) | 0.001 |
| Biceps skinfold (mm) | 0.059 | 1.06 (1.02, 1.11) | 0.009 |
| Triceps skinfold (mm) | 0.062 | 1.06 (1.02, 1.11) | 0.008 |
| Subscapular skinfold (mm) | 0.081 | 1.08 (1.04, 1.14) | 0.001 |
| Suprailiac skinfold (mm) | 0.024 | 1.02 (0.97, 1.08) | 0.36 |
| Sum of skinfolds (mm) | 0.059 | 1.06 (1.02, 1.11) | 0.005 |
| **Body composition (DXA)** |  |  |  |
| Fat mass (kg) | 0.104 | 1.11 (1.03, 1.19) | 0.005 |
| Fat mass index (kg/m^2^) | 0.104 | 1.11 (1.04, 1.19) | 0.003 |
| Fat % | 0.072 | 1.07 (1.02, 1.13) | 0.009 |
| Android fat (kg) | 0.099 | 1.10 (1.02, 1.20) | 0.02 |
| Gynoid fat (kg) | 0.059 | 1.06 (1.00, 1.12) | 0.04 |
|  |  |  |  |
| **MODEL 2 ^5^** |  |  |  |
| **Anthropometry** |  |  |  |
| Body mass index (kg/m^2^) | 0.021 | 1.02 (0.99, 1.04) | 0.06 |
| Biceps skinfold (mm) | 0.060 | 1.06 (1.01, 1.12) | 0.02 |
| Triceps skinfold (mm) | 0.047 | 1.05 (0.99, 1.11) | 0.09 |
| Subscapular skinfold (mm) | 0.076 | 1.08 (1.02, 1.14) | 0.01 |
| Suprailiac skinfold (mm) | 0.004 | 1.00 (0.94, 1.07) | 0.89 |
| Sum of skinfolds (mm) | 0.050 | 1.05 (1.00, 1.11) | 0.05 |
| **Body composition (DXA)** |  |  |  |
| Fat mass (kg) | 0.078 | 1.08 (0.99, 1.18) | 0.08 |
| Fat mass index (kg/m^2^) | 0.084 | 1.09 (1.00, 1.18) | 0.04 |
| Fat % | 0.063 | 1.07 (1.00, 1.14) | 0.06 |
| Android fat (kg) | 0.068 | 1.07 (0.97, 1.18) | 0.18 |
| Gynoid fat (kg) | 0.037 | 1.03 (0.97, 1.11) | 0.29 |
|  |  |  |  |

^1^ Differences in adiposity outcomes between maternal allocation groups were analyzed using multiple linear regression, adjusting for the same factors as shown in Table 5 of the main manuscript. All the outcomes were right skewed and were logged for the analysis. P indicates the significance of differences between intervention and control groups.

^2^ The regression coefficient represents the difference in the logged outcome between the control and intervention groups.

^3^ Because outcomes were log-transformed, to derive the difference between intervention and control groups the regression coefficient is first exponentiated (anti-logged): values become 1.11 for Model 1 and 1.09 for Model 2 and these values indicate the multiplicative difference between control and intervention groups; for example a value of 1.11 in the difference column means that the outcome was 11% higher in the intervention group than in the control group.

^4^ Model 1 is adjusted for maternal age, height, pre-pregnant BMI and parity, socio-economic status score, and the child’s age

^5^ Model 2 is adjusted for the same variables as Model 1, and additionally for the child’s birth weight and gestational age at birth. DXA: Dual-energy absorptiometry.
